# Supplementary material for: Uncovering Predictive Factors and Interventions for Restoring Microecological Diversity after Antibiotic Disturbance
Source: Nutrients. 2023 Sep 10;15(18):3925. doi: 10.3390/nu15183925 (PMC10536327; doi:10.3390/nu15183925)
Supplement: Supplementary file 1 [file nutrients-15-03925-s001.zip › Supplementary_figures.pdf]

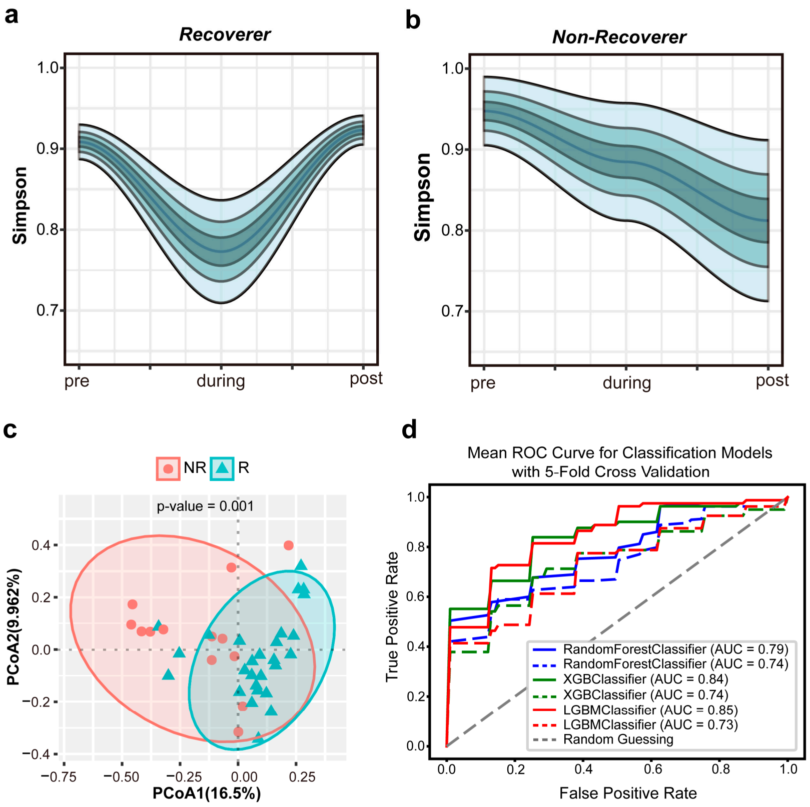

Supplementary Figure S1. Gut microbiome recovery profiles and ROC curves in several classifiers of the Verification dataset. (**a**,**b**) Density plots showing the two different recovery profiles for microbial diversity (Shannon) that were observed in the antibiotic treatment cohorts (DK1, DK2, US). (**c**) PCoA figure showing the distribution of Bray-Curtis distances for post-antibiotic gut microbiomes for recoverers and non-recoverers. (p = 0.001, PERMANOVA, Adonis) (**d**) The ROC curves and areas under the curves (AUC) for three models (Random Forest, XGBoost, LightGBM) of p-RABs (solid lines) and RABs (dotted lines) across all time including pre, during, and after-antibiotic treatment.

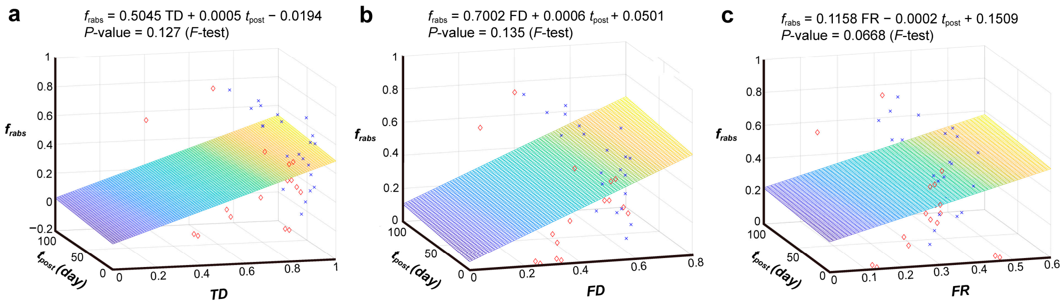

Supplementary Figure S2. Multiple linear regression showed the effect of Taxonomic Diversity (TD), Functional Diversity (FD), and Functional Redundancy (FR) on recovery after antibiotic disturbance. (**a**) taxonomic diversity (TD) that is the Gini-Simpson index; (**b**) Rao's quadratic entropy calculated as functional diversity (FD); and (**c**) functional redundancy (FR = TD − FD) of gut microbiota, associated with days after antibiotic treatment and the relative abundance ratio of RABs (obtained by one-sided Wilcoxon test). Blue nodes represent recoverers and red nodes represent non-recoverers. The p values were calculated from the F-test.

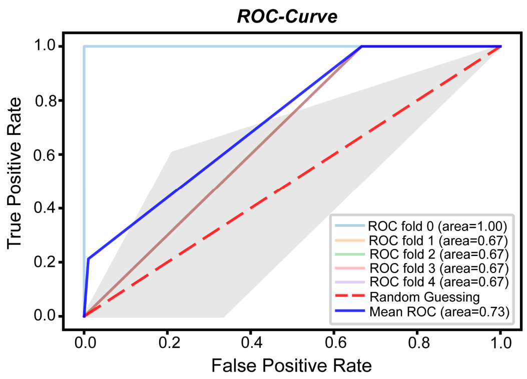

Supplementary Figure S3. ROC curves of logistic regression. The five ROC curves and their average ROC curve were obtained from a 5-fold cross-verified logistic regression equation.

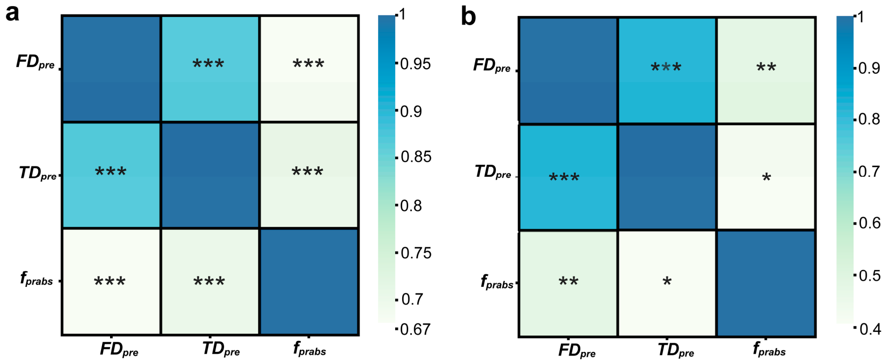

Supplementary Figure S4. Pearson and Spearman correlation between FD, TD, and f_{prabs} at pre-antibiotic treatment. (**a**) Pearson correlation showing the correlations between FD with TD, FD with f_{prabs}, and TD with f_{prabs} are all significant. (* means $p < 0.05$, ** means $p < 0.01$, *** means $p < 0.001$) (**b**) Spearman correlation showing the correlations between FD with TD, FD with f_{prabs}, and TD with f_{prabs} are also significant.

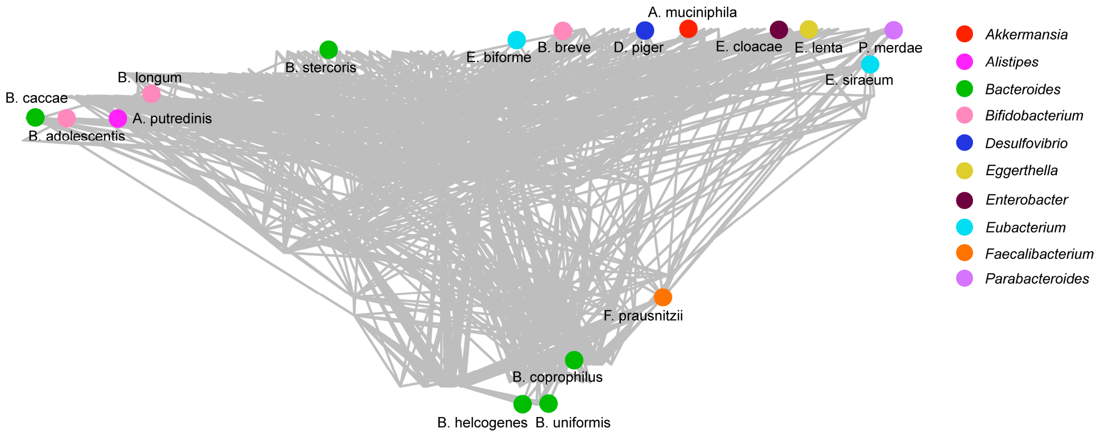

Supplementary Figure S5. The p-RABs in the microbial food web. A graph showing the network structure of microbial dependencies is inferred using an association rule mining approach, where an edge from species A to species B indicates that the presence of A is required to have B in the community. Nodes are ordered from the bottom to the top such that species at the bottom have more outgoing edges than incoming edges ("primary species"), while species at the top have more incoming edges than outgoing edges (metabolically supported by primary species). P-RABs (highlighted in different colors based on the genus they belong to) were observed either at the bottom or the top of the graph.

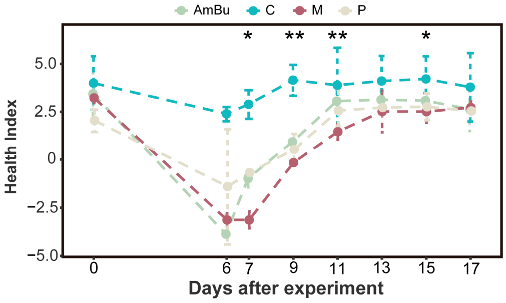

Supplementary Figure S6. Changes of the health index-GMHI in the mouse experiment. Observed changes in the health index in different groups of mice across time. The * $p < 0.05$ and ** $p < 0.01$ for time points where the AmBu group significantly differs from the model group (one-sided Wilcoxon test). P is the positive group (BtBa), C is the negative control group (1× PBS), M is the model group (antibiotic without intervention) and AmBu is the treatment group (AmBu).

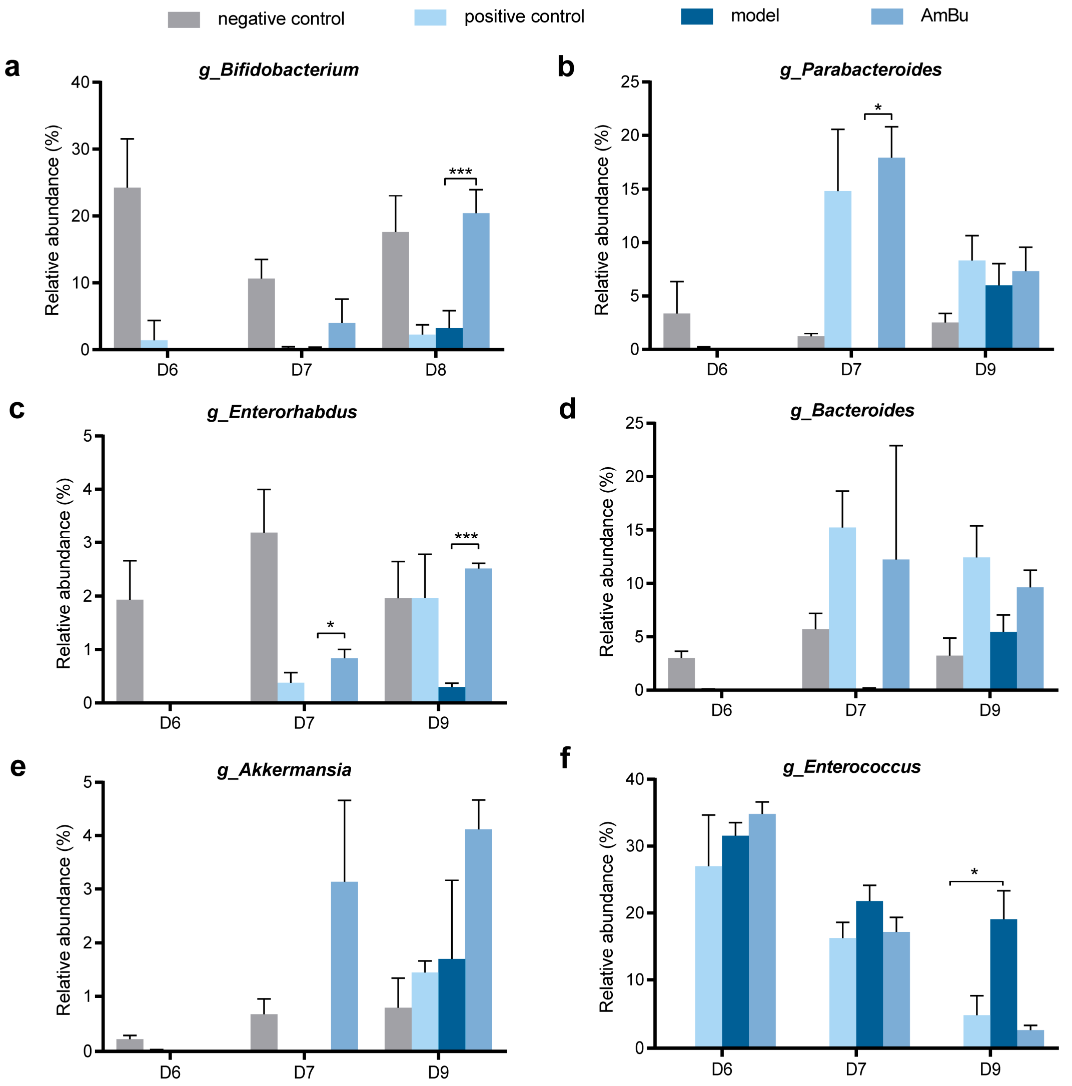

Supplementary Figure S7. Changes of the bacteria after antibiotic treatment in the mouse experiment. Different colors represent the relative abundance of different groups at different time points (D6: one day after antibiotics; D7: one day after intervention; D9: three days after intervention) after antibiotics. (**a**) *g_Bifidobacterium*, (**b**) *g_Parabacteroides*, (**c**) *g_Enterorhabdus*, (**d**) *g_Bacteroides*, and (**e**) *g_Akkermansia* that decreased after antibiotic treatment recovered with gavaged *Akkermansia muciniphila* and *Bacteroides uniformis*, (**f**) *g_Enterococcus* that significantly decreased and is close to negative control group when given intervention (* means $p < 0.05$, *** means $p < 0.001$).
